# Metabolic Flux Analysis Reveals Entner–Doudoroff Pathway Dominance in Heterotrophic Deep‐Sea Bacterial Isolates

**DOI:** 10.1111/1758-2229.70379

**Published:** 2026-06-12

**Authors:** Yuxue Yang, Yue Wu, Keni Ma, Yue Li, Junwei Cao, Yuli Wei, Ruilian Yao, Weichao Wu

**Affiliations:** ^1^ College of Oceanography and Ecological Science Shanghai Ocean University Shanghai China; ^2^ International Research Center for Marine Biosciences at Shanghai Ocean University, Ministry of Science and Technology Shanghai China; ^3^ State Key Laboratory of Microbial Metabolism, Joint International Laboratory on Metabolic and Developmental Sciences, School of Life Sciences and Biotechnology Shanghai Jiao Tong University Shanghai China

**Keywords:** ^13^C metabolic flux analysis, anaplerotic reactions, central carbon metabolic flux, deep‐sea bacteria

## Abstract

Deep‐sea microorganisms adapt to extreme conditions by diverse metabolic strategies, yet their intracellular carbon fluxes remain largely unexplored. Here, we investigated the central carbon metabolic fluxes of four bacterial strains isolated from Pacific Ocean sediments and deep waters, including *Stutzerimonas marianensis* PS1, *Shewanella piezotolerans* WP3, *Georhizobium profundi* WS11 and *Parasedimentitalea marina* W43. Using ^13^C metabolic flux analysis with multiple ^13^C‐labelled glucose isotopologues, all strains preferentially degraded glucose via the Entner–Doudoroff pathway (EDP) with fluxes accounting for 66.7%–94.0% of total glycolytic flux, indicating that EDP is a conserved and dominant glycolytic route among deep‐sea heterotrophs. *S. piezotolerans* WP3 uniquely exhibited substantial flux through both oxidative and non‐oxidative branches of the pentose phosphate pathway, suggesting heightened precursor and redox demands. The strains also displayed diverse anaplerotic strategies, engaging either phosphoenolpyruvate or pyruvate carboxylation to replenish tricarboxylic acid cycle intermediates and enhance carbon utilisation efficiency. Oxidative stress assays further revealed a link between intracellular energy status and tolerance to hydrogen peroxide. Collectively, these findings provide comparative fluxomic evidence for central carbon metabolism in deep‐sea bacteria and highlight metabolic traits that support survival in carbon‐limited, high‐pressure marine environments.

## Graphical abstract

Four deep‐sea bacterial strains, isolated from sediments at the water depths of 1–10 km, exhibited diverse metabolic flux patterns in central carbon metabolism as well as differing antioxidative responses.

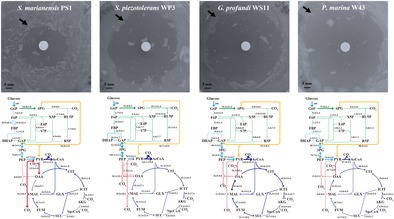

## 1 Introduction

The deep sea is one of the most extensive habitats on Earth and is characterised by low temperatures, high hydrostatic pressure and limited nutrient availability (Jian et al. 2016). Such extreme conditions drive microorganisms to develop diverse metabolic activities, species‐specific adaptations and unique physiological mechanisms (Orcutt et al. 2011). Deep‐sea microbes play a crucial role in global biogeochemical cycles (e.g., carbon, nitrogen and phosphorus), thereby contributing significantly to ecosystem stability. Compared with terrestrial bacteria, deep‐sea microorganisms exhibit distinct growth regulation and stress‐defence strategies (Hoshino et al. 2020; Jørgensen and Marshall 2016). For example, some species adapt to high pressure or low temperature by modifying membrane permeability and fluidity, reconfiguring metabolic pathways or enhancing antioxidative responses (Cario et al. 2015; Jian et al. 2016; Qin et al. 2021). These observations suggest highly dynamic intracellular metabolic processes that support microbial survival in the deep ocean. Understanding these adaptive metabolic mechanisms is increasingly relevant to studies of microbial evolution, deep‐sea ecology and industrial biotechnology (Gauthier et al. 2021; Xiao et al. 2025; Zhang et al. 2024). Thus, elucidating the intracellular anabolic and catabolic routes of deep‐sea microbes is essential for understanding their adaptation strategies.

Intracellular metabolic fluxes represent the integrated outcome of enzyme activities, regulatory networks and environmental stressors, offering a comprehensive view of cellular energy flow and carbon transformation (Bore et al. 2017; Law et al. 2022). Metabolic flux analysis (MFA) quantifies the direction and magnitude of carbon flow through metabolic networks and is a powerful approach for determining substrate utilisation preferences and key pathway activities. In heterotrophic bacteria, glucose is metabolised through upper glycolytic pathways and the downstream tricarboxylic acid (TCA) cycle. Upper glycolysis encompasses three major routes: the Embden–Meyerhof–Parnas pathway (EMPP), the Entner–Doudoroff pathway (EDP) and the pentose phosphate pathway (PPP). The TCA cycle can be replenished through anaplerotic routes, such as oxaloacetate or malate formation, or through the glyoxylate shunt. Together, these interconnected pathways form the central carbon metabolism (CCM), which supports both energy production and biosynthesis (Noor et al. 2010; Thomas et al. 2019).

To date, most metabolic flux studies have focused on terrestrial model organisms, such as *Escherichia coli* , *Bacillus subtilis* and *Saccharomyces cerevisiae* (Crown et al. 2015; Dauner et al. 2001; Nidelet et al. 2016). These species predominantly utilise the EMPP and PPP during glucose catabolism, although the relative fluxes through each pathway vary depending on environmental conditions (Fuhrer et al. 2005; Nikel et al. 2015, 2021). For example, *E. coli* directs ~80% of glucose through the EMPP, while *S. cerevisiae* directs up to 95% (Fuhrer et al. 2005; Nidelet et al. 2016). *B. subtilis* channels more flux through the PPP than *E. coli* , likely due to increased NADPH demand for biosynthetic processes such as fatty acid, peptide and riboflavin production (Bremer et al. 2023; Dauner and Sauer 2001; Yin et al. 2024). In contrast, EDP activity is minimal or absent in these organisms but becomes dominant in species such as *Pseudomonas* due to the lack of phosphofructokinase (Nikel et al. 2015; Wijker et al. 2019; Wu et al. 2020).

These findings have motivated growing interest in understanding central carbon flux patterns in marine bacteria. Klingner et al. (2015) performed the first large‐scale MFA study of marine isolates from coastal seawater, revealing a surprisingly widespread reliance on the EDP. In addition, marine strains appear to exhibit two major flux patterns, with some strains predominantly utilising the EMPP and others favouring the EDP. Conversely, a more recent MFA study on microbial communities in coastal sediments suggested that EMPP may be favoured in benthic environments (Hutchinson et al. 2024). The discrepancy between seawater isolates and sediment communities may reflect: (1) differences in environmental conditions between the water column and sediments or (2) metabolic interactions and substrate exchange within microbial communities. To disentangle these effects, MFA of pure sediment‐derived isolates is required.

MFA, as a branch of fluxomics within systems biology, provides quantitative and mechanistic insight into cellular metabolism by integrating isotopic labelling data with metabolic network models (Emwas et al. 2022; Long and Antoniewicz 2019; Mishra and Kumar 2024; Yao 2021). Although this approach has been widely used in metabolic engineering and biomedical research, it has rarely been applied to deep‐sea bacteria (Long and Antoniewicz 2019; Yuan et al. 2008; Zamboni et al. 2009).

In this study, we performed ^13^C‐MFA on four bacterial strains isolated from sediments and deep waters of the Pacific Ocean at depths ranging from 1 to 10 km. Our goal was to characterise their central carbon metabolic flux distributions and to assess how these flux patterns relate to taxonomic identity and potential adaptive traits. By elucidating the intracellular metabolic strategies of these deep‐sea microbes, this work advances our understanding of microbial adaptation in extreme environments and highlights metabolic features with potential biotechnological applications, such as cold‐active or high‐pressure‐tolerant pathways.

## 2 Materials and Methods

### 2.1 Bacterial Strains

For this study, four deep‐sea bacterial strains were cultivated (see Table 1) including: *Stutzerimonas marianensis* PS1 (MCCC 1K05112) isolated from Mariana Trench sediments (11.33° N, 142.2° E) (Rudra and Gupta 2023; Yang et al. 2022); *Shewanella piezotolerans* WP3 (MCCC 1A01966) isolated from the sediments of the Pacific Ocean (water depth of 1914 m) (8°00′11′′ N, 142°30′08′′ E) and obtained from the Marine Culture Collection of China (Xiao et al. 2007); *Georhizobium profundi* WS11 (MCCC 1K03498) derived from New Britain Trench sediments (water depth of 4524 m) (6.7° S, 149.8° E) (Cao et al. 2020); *Parasedimentitalea marina* W43 (MCCC 1K03532), collected from New Britain Trench seawater (water depth of 4000 m) (7.0° S, 149.8° E) (Ding et al. 2019; Zhang et al. 2022). The 16S rRNA and whole‐genome sequences of all strains are available in the NCBI (http://www.ncbi.nlm.nih.gov/).

**TABLE 1:** Deep‐sea bacterial strains used in this work.

Strains	Class	Family	Accession numbers in NCBI		References
			Genome	16S rRNA	
*S. marianensis* PS1	*Gammaproteobacteria*	*Pseudomonadaceae*	JALGRD000000000	MZ670768	Rudra and Gupta (2023) and Yang et al. (2022)
*S. piezotolerans* WP3	*Gammaproteobacteria*	*Shewanellaceae*	CP000472	AJ551090	Xiao et al. (2007)
*G. profundi* WS11	*Alphaproteobacteria*	*Rhizobiaceae*	CP032509	MG822857	Cao et al. (2020)
*P. marina* W43	*Alphaproteobacteria*	*Roseobacteraceae*	CP033219–CP033223	MK084778	Ding et al. (2019) and Zhang et al. (2022)

### 2.2 Strain Cultivation

The laboratory‐conserved strains in glycerol (−80°C) were inoculated into 20 mL of 2216E liquid medium (Bonnet et al. 2020; Patrick 1978) at the respective optimal temperatures (e.g., PS1 at 35°C, WP3 and W43 at 20°C and WS11 at 28°C) until the late mid‐logarithmic stage. The cultures were then washed with 0.9% NaCl three times and transferred into optimised LMO‐812 medium for liquid incubation with inoculum levels of 1% or 5%. All incubations were carried out under aerobic conditions.

The optimised LMO‐812 medium contained two basic solutions A and B with each 500 mL. Solution A contained the following chemicals (quantities per litre) at pH 7.5: 52.0 g NaCl, 8.0 g Na_2_SO_4_, 0.6 g NH_4_Cl, 0.2 g KH_2_PO_4_ and 1.0 g KCl. Solution B had (quantities per litre) 10.0 g MgCl_2_·6H_2_O, 2.8 g CaCl_2_·2H_2_O. The following components (quantities per 100 mL of final medium) were sterilised separately and then added into the basic medium: 2 mL of 8.4 g/L NaHCO_3_, 100 μL of vitamin solution, 100 μL of trace metal solution. The vitamin solution included the following substrates: 6 μM thiamine, 800 nM niacin, 425 nM pantothenic acid, 500 nM pyridoxine, 4 nM biotin, 4 nM folic acid, 700 pM cobalamin, 6 μM myo‐inositol and 60 nM 4‐aminobenzoic acid. The trace metal solution included 117 nM FeCl_3_·6H_2_O, 9 nM MnCl_2_·4H_2_O, 800 pM ZnSO_4_·7H_2_O, 500 pM CoCl_2_·6H_2_O, 300 pM Na_2_MoO_4_·2H_2_O, 1 nM Na_2_SeO_3_ and 1 nM NiCl_2_·6H_2_O.

In all experiments, sterilised glucose via 0.22 μm filters was supplemented as the sole carbon source at the concentrations for minimum microbial growth as reported (Cao et al. 2020; Chen et al. 2022; Ding et al. 2019; Yang et al. 2022; Zhang et al. 2022). Specifically, 5 mM glucose was used for strain PS1, 20 mM for strain WP3 and 10 mM for strain WS11 and strain W43. The applied temperatures corresponded to the optimal conditions identified in the previous isolation procedure: 35°C for PS1, 20°C for WP3, 28°C for WS11 and 20°C for W43. For ^13^C‐labelled incubation experiments, the parallel labelling strategy was suggested to improve the precision of MFA (Crown et al. 2015; Rahim et al. 2022; Young 2014). Hereby we used three glucose isotopologues including 1‐^13^C‐glucose (99% purity), a mixture of U‐^13^C_6_‐glucose (98% purity) and natural glucose (20:80, wt/wt), and a mixture of U‐^13^C_6_‐glucose and 1‐^13^C‐glucose (20:80, wt/wt). All glucose isotopologues were purchased from Cambridge Isotope Laboratories (US). The applied concentrations of ^13^C‐glucose isotopologues for four strains were the same as the precultures.

### 2.3 Measurement of Glucose, ATP, NAD(H) and NADP(H)

A 100 μL aliquot of the supernatant collected at the sampling time was filtered through a 0.22 μm filter (PES, 25 mm), and glucose concentration was determined using a glucose assay kit based on the glucose oxidase‐peroxidase method (Boxbio, AKSU001, China) (Figure S1A), following instructions by the manufacturer.

Intracellular ATP, NAD^+^/NADH and NADP^+^/NADPH levels were measured during the late exponential phase. Cells were harvested by centrifugation at 12,000*g* for 10 min at 4°C. For ATP analysis, approximately 5 × 10^6^ cells were collected, whereas for NAD^+^/NADH and NADP^+^/NADPH measurements, culture volumes corresponding to 1.25 OD_600_ units were used. In addition, these metabolites were measured in *B. subtilis* as a reference organism for comparative analysis, following previously reported protocol (Klingner et al. 2015). Concentrations of ATP, NAD^+^/NADH and NADP^+^/NADPH were determined using enzymatic reactions with commercial kits according to the instructions by manufacturer recommended. The kits were BL852A (Biosharp, China), S0175 and S0179 (both from Beyotime, China) (Figure S1B,C), respectively. The method details are listed in the Supporting Information.

### 2.4 Antioxidant Activity Test

This test was performed as the method by Xie et al. (2018). The cultures were grown on 2216E medium until mid‐log phase (OD_600_ = 0.5), and then 200 μL cultures were transferred evenly on the solid optimised LMO‐812 medium (1.5% agar and 20 mM glucose) using an L‐shaped spreader. Before transferring, the cultures were washed three times with optimised LMO‐812 to remove the original medium. Twenty microlitres of 6% hydrogen peroxide solution was put on the blank filter discs until air dryness, and the filter was put onto the culture pots. Oxidative stress resistance was assessed by measuring the responsive area (diameter in cm) until the inhibition area stabilised.

### 2.5 Proteinogenic Amino Acid Analysis by GC–MS

**FIGURE 1:**
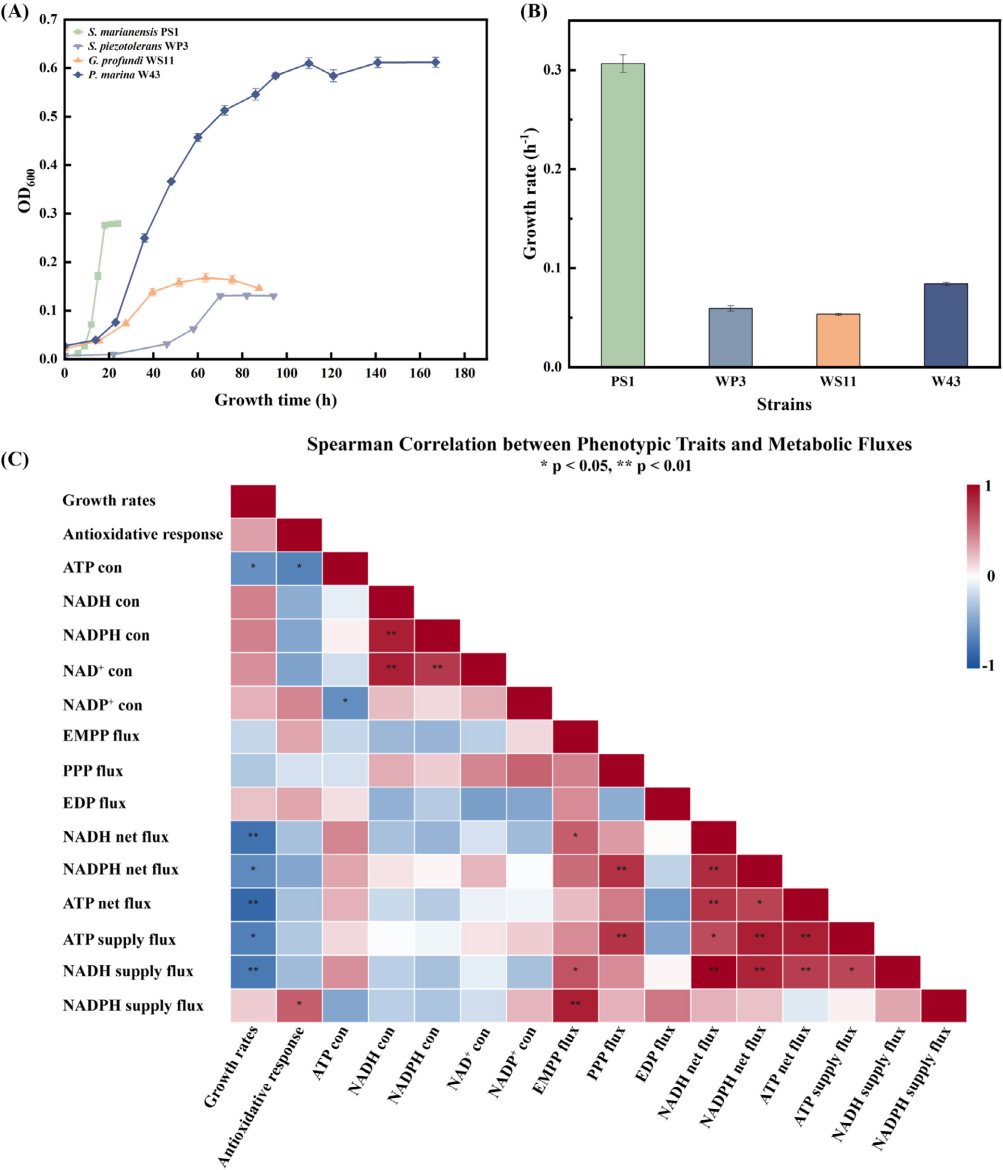
Growth characteristics of the four strains and their correlations with intracellular metabolic fluxes and metabolite levels. (A) Growth curves of the four strains cultivated with glucose as the sole carbon source. (B) Maximum specific growth rates estimated from OD_600_ measurements during the exponential phase. (C) Spearman correlation analysis between growth rates and the fluxes or intracellular concentrations of metabolites and central carbon metabolic pathways. ATP con, NADPH con and NADH con represent the intracellular concentrations of ATP, NADPH and NADH measured at the late exponential phase. ATP supply flux, NADPH supply flux and NADH supply flux represent the fluxes of ATP, NADPH and NADH calculated from the relative fluxes of EMPP, PPP, EDP and TCA cycles. ATP net flux, NADPH net flux and NADH net flux represent the fluxes of ATP, NADPH and NADH calculated from the relative fluxes of all reactions in the metabolic model.

One mL of culture at late exponential phase was used for the amino acid analysis and the subsequent MFA. In general, cells at the late exponential phase are assumed to have reached both metabolic and isotopic steady‐state conditions (Long and Antoniewicz 2019; Yuan et al. 2008). Considering the different growth status, the corresponding harvest time for PS1, WP3, WS11 and W43 were 16, 65, 45 and 85 h, respectively, with OD_600_ values of 0.25, 0.15, 0.18 and 0.57 (Figure 1). The lyophilised bacterial pellets were transferred into clean FEP tubes, followed by the addition of 500 μL of 6 N HCl. The samples were then hydrolysed at 110°C for 16 h (Long and Antoniewicz 2019). After cooling to room temperature, the hydrolysates were centrifuged at 14,000*g* for 5 min at ambient temperature. The resulting supernatant was carefully transferred to 2 mL glass vials and evaporated to dryness either under N_2_ at 65°C or 110°C. The dried residues were subsequently derivatised with 50 μL of pyridine and 50 μL of MTBSTFA containing 1% (wt/wt) TBDMCS at 70°C for 40 min. The derivatised samples were directly analysed by a gas chromatography–mass spectrometry (GC–MS 7890B/5977A, Agilent, US) within 24 h to ensure optimal stability of the derivatives. The samples were injected at 250°C with split mode (ratio of 1:10) and then separated on an HP‐5 ms chromatographic column (30 m × 250 μm × 0.25 μm, Agilent). The oven temperature was programmed as follows: initialised at 80°C for 2 min, and then ramped to 280°C at 7°C/min for 20 min (Long and Antoniewicz 2019). The MS conditions were set up as follows: transfer line temperature at 280°C, ion source temperature at 230°C, quadrupole temperature at 150°C and EI energy at 70 eV. The full scan mode was used to monitor the fragmental abundance.

### 2.6 GC–MS Data Analysis and MFA

#### 2.6.1 GC–MS Data Analysis

Qualitative analysis of amino acids was performed using MassHunter software with the NIST14.L database and confirmed by authentic standards. The selected mass fragmental abundance of amino acids was integrated including (M‐15)^+^, (M‐57)^+^, (M‐85)^+^, (M‐159)^+^ and (f302)^+^, where M and f302 refer to the molecular and fragmental mass *m*/*z* 302, respectively (Schmitz et al. 2017; You et al. 2012). The detailed information of selected fragments for amino acids in this study is listed in Supporting Information. The peak area of each mass isotopomer for a specific fragment was converted to fractional abundance as mass distribution vectors (MDVs). The ^13^C‐labelled mass isotopomer was corrected for the natural ^13^C contribution (1.07%) using the correction algorithm by Nanchen et al. (2007). The corrected MDVs of amino acid fragments were used for the following flux analysis with the following two approaches.

#### 2.6.2 Intracellular MFA

Prior to metabolic analysis, a metabolic network was reconstructed based on genome annotation using KEGG and other databases (Cao et al. 2020; Xiao et al. 2007; Yang et al. 2022; Yao 2021; Zhang et al. 2022). In total, 67 biochemical reactions were included, covering six major central carbon metabolic pathways (CCMPs): EMPP, PPP, EDP, TCA, anaplerotic reactions and glyoxylate shunt. Central metabolites were assumed to be at metabolic (pseudo) steady state. Co‐factors (e.g., ATP, NADH and NADPH) were not explicitly balanced in the model (four reactions), while acetate and CO_2_ were treated as non‐balanced metabolites (two reactions). It should be noted that genome annotation indicated that certain biochemical reactions were inactive in specific strains. For example, the reaction phosphoenolpyruvate (PEP) + CO_2_ → oxaloacetate (OAA) was absent in strains WS11 and W43. These strain‐specific differences were incorporated into the model by constraining the corresponding reactions accordingly. For biomass equation in the model, we assumed the sample biomass composition (protein, RNA, DNA and lipid contents) as *Pseudomonas putida* (Czajka et al. 2022) since the precise biomass composition was not estimated in this study. A complete list of biochemical reactions is provided in Table S1. MFA was performed using two complementary approaches.

First, simple equations based on the MDVs of alanine (Equations (1), (2), (3)), obtained from the incubation with 1‐^13^C‐glucose, were used to estimate the relative ratios of three glycolytic pathways (i.e., EMPP, PPP and EDP; Klingner et al. 2015). This approach enabled a direct and quick comparison of metabolic flux patterns in the marine strains examined in this study with those reported by Klingner et al. (2015), using an identical approach. Alanine was selected for metabolic flux estimation because its precursor pyruvate lies at a key metabolic node where the fluxes from the EMPP, PPP and EDP converge. In addition, Klingner et al. (2015) characterised metabolic flux patterns across a large set of marine strains, applying a comparable approach which facilitates meaningful comparison and evaluation of potential differences among strains.

(1) $$f_{\mathrm{PPP}}=2\left(M_{0,{\mathrm{Ala}}_{123}}-0.5\right)$$

(2) $$\begin{array}{c} f_{\text{EMPP}}=-2\left(M_{0,{\mathrm{Ala}}_{23}}-1\right) \end{array}$$

(3) $$\begin{array}{c} f_{\mathrm{EDP}}=1-f_{\mathrm{PPP}}-f_{\text{EMPP}} \end{array}$$

where *f*_PPP_, *f*_EMPP_ and *f*_EDP_ refer to the relative flux through the pathways of PPP, EMPP and EDP. M_0,Ala123_ and M_0,Ala23_ are the relative abundance of monoisotopic fragmental alanine C1–C3 (i.e., *m*/*z* = 260) and alanine C2–C3 (i.e., *m*/*z* = 232), respectively.

Secondly, to improve the high resolution of metabolic flux estimates such as TCA cycle, anaplerotic reactions and glyoxylate shunt, the second approach was based on complementary modelling approaches integrating parallel ^13^C‐treatments with multiple ^13^C‐labelled glucose isotopologues by the ^13^C‐MFA package INCA2.3 (Rahim et al. 2022). This method aims to reveal more details on the intracellular biochemical reactions and explore the metabolic diversity among these strains.

Metabolic fluxes were estimated by minimising the variance‐weighted sum of squared residuals (SSR) between the experimentally measured and model predicted mass isotopomer distributions using non‐linear least‐squares regression combining the parallel ^13^C‐labelling results including 1‐^13^C‐glucose (99% purity), a mixture of U‐^13^C_6_‐glucose (98% purity) and natural glucose (20:80, wt/wt) and a mixture of U‐^13^C_6_‐glucose and 1‐^13^C‐glucose (20:80, wt/wt).

### 2.7 Statistical Analysis

Flux estimation was repeated at least 10 times using random initial values to ensure convergence to the global optimum. The goodness‐of‐fit between the simulated and experimental MDVs was evaluated using the sum of SSR and assessed statistically with a *χ* ^2^ test. Specifically, the minimised SSR was compared with the 95% confidence interval of the *χ* ^2^ distribution based on the corresponding degrees of freedom (Leighty and Antoniewicz 2013). All statistical analyses were performed using the INCA software package (Rahim et al. 2022). The SSR values for experimental (Figure S2) and simulated MDVs, along with the full set of raw output results, are provided in the Supporting Information.

To investigate the relationships between phenotypic parameters (e.g., growth rate and oxidative stress tolerance) and intracellular energy‐related metabolites and fluxes (ATP, NAD(P)H and NAD(H)), Spearman's rank correlation analysis was performed. The supply fluxes of ATP, NADPH and NADH were calculated based on the EMPP, EDP, PPP and TCA cycle pathways, whereas the net fluxes were derived from all reactions included in the metabolic model. Oxidative stress tolerance was assessed using a disk diffusion assay, in which the diameter of the inhibition zone was used as a proxy. As larger inhibition zones indicate lower oxidative stress tolerance, the negative of the inhibition zone diameter was used to represent antioxidative performance in the correlation analysis.

## 3 Results and Discussion

### 3.1 Distinct Growth and Substrate Utilisation/Production Rates

Different growth rates among these four microbial strains were clearly observed given the identical sole carbon source, i.e., glucose (Figure 1). Such diverse growth rates reveal dynamic metabolic processes within bacterial development from initial adaptation to stationary phase (Cuevas and Edwards 2017; Mytilinaios et al. 2012). Since these microbes were isolated from the surface sediment and water of different depths (1–10 km) with different environments (e.g., hydrostatic pressures, organic matter supplies and oxygen consumption) (Glud et al. 2013; Jørgensen et al. 2022; Xu et al. 2021), these environmental variations could result in the distinct genotypes, further shaping various growth rates of microbes. In this study, the growth curves of all four strains exhibit typical sigmoidal characteristics also called ‘S‐curve’ (Figure 1A). The growth curve shows large heterogeneity in the duration of the exponential phase with 10, 47, 17 and 15 h for strains PS1, WP3, WS11 and W43, respectively. Accordingly, the maximum specific growth rates are 0.307 ± 0.009 h^−1^, 0.059 ± 0.003 h^−1^, 0.054 ± 0.001 h^−1^ and 0.084 ± 0.002 h^−1^ for the respective strains (Figure 1B). It is important to note that each strain was cultivated at its own optimal isolation temperature (35°C, 20°C, 28°C and 20°C, respectively). Therefore, the absence of a strong correlation between temperature and growth rate is expected, likely reflecting species‐specific metabolic capacity, enzyme efficiency and regulatory strategies. Given the identical substrate, the diverse growth rates for these bacterial strains imply various intracellular catabolic and anabolic reactions (Gonzalez and Aranda 2023; Lipson 2015). Consequently, differences in growth rates are more likely attributed to intrinsic metabolic and physiological differences among the strains rather than temperature effect (Chen and Nielsen 2019).

The energy‐relevant substrates (e.g., ATP, NADH and NADPH) were analysed to verify microbial growth rates and the energy supply at the late exponential phase. The varying production of the above‐mentioned substrates at harvest indicated differential catabolic activities during the growth phase (Sun et al. 2023). However, *S. marianensis* PS1 with the highest growth did not yield the highest amounts of ATP, NAD^+^/NADH and NADP^+^/NADPH (Table 2). Furthermore, a strong correlation was observed only between microbial growth rates and the fluxes or intracellular concentrations of energy‐related metabolites (e.g., ATP and NADH) (*p* < 0.05; Figure 1C). This pattern may reflect the maintenance of these energy‐associated metabolites at dynamic steady‐state levels during exponential growth, resulting from the balance between energy production and consumption across different CCMPs (Mu et al. 2024; Sun et al. 2023; van Niel et al. 2017).

**TABLE 2:** Substrate production and consumption rates among the four deep‐sea bacterial strains.

Strains	Glucose consumption rate (μmol/h)	Content			Biomass yield (CDW [g]/g of glucose)
		ATP (pmol/10^4^ cell)	NAD^+^/NADH (μM)	NADP^+^/NADPH (μM)	
*S. marianensis* PS1	3.35 ± 1.28	0.03 ± 0.01	0.32 ± 0.10/0.15 ± 0.02	1.69 ± 0.58/1.16 ± 0.36	0.55 ± 0.04
*S. piezotolerans* WP3	0.77 ± 0.09	0.03 ± 0.00	1.16 ± 0.19/0.19 ± 0.05	3.96 ± 0.36/0.83 ± 0.26	0.20 ± 0.02
*G. profundi* WS11	0.74 ± 0.15	0.09 ± 0.01	0.10 ± 0.06/0.12 ± 0.02	0.77 ± 0.09/0.46 ± 0.10	0.49 ± 0.02
*P. marina* W43	0.89 ± 0.24	0.07 ± 0.01	2.12 ± 0.10/0.32 ± 0.10	0.68 ± 0.54/2.97 ± 0.48	1.07 ± 0.16
*B. subtilis* 168	n.d.	0.27 ± 0.03	6.42 ± 0.42/0.34 ± 0.04	2.17 ± 0.48/3.08 ± 0.01	n.d.

*Note:* Values are given as means ± standard deviations.

Abbreviations: CDW, cellular dry weight; n.d., not determined.

### 3.2 ED Pathways as an Important Glycolytic Route for Deep‐Sea Microbes

**FIGURE 2:**
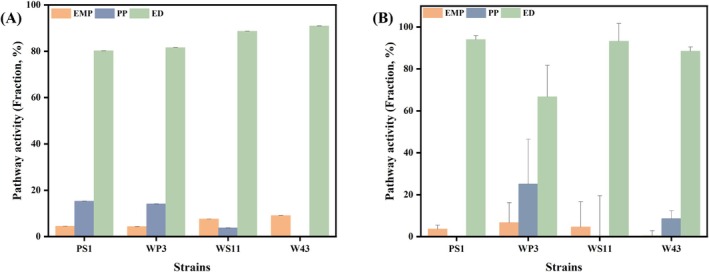
Comparison of the relative metabolic flux of three glycolytic pathways for two approaches. (A) The fluxes were estimated by the mass isotopomer distribution vector of alanine fragments based on incubation treatment of 1‐^13^C‐glucose as Klingner et al. (2015). (B) It was estimated with the three parallel experiments with different ^13^C‐labelled glucose isotopologues. See Section 2 for more details.

**FIGURE 3:**
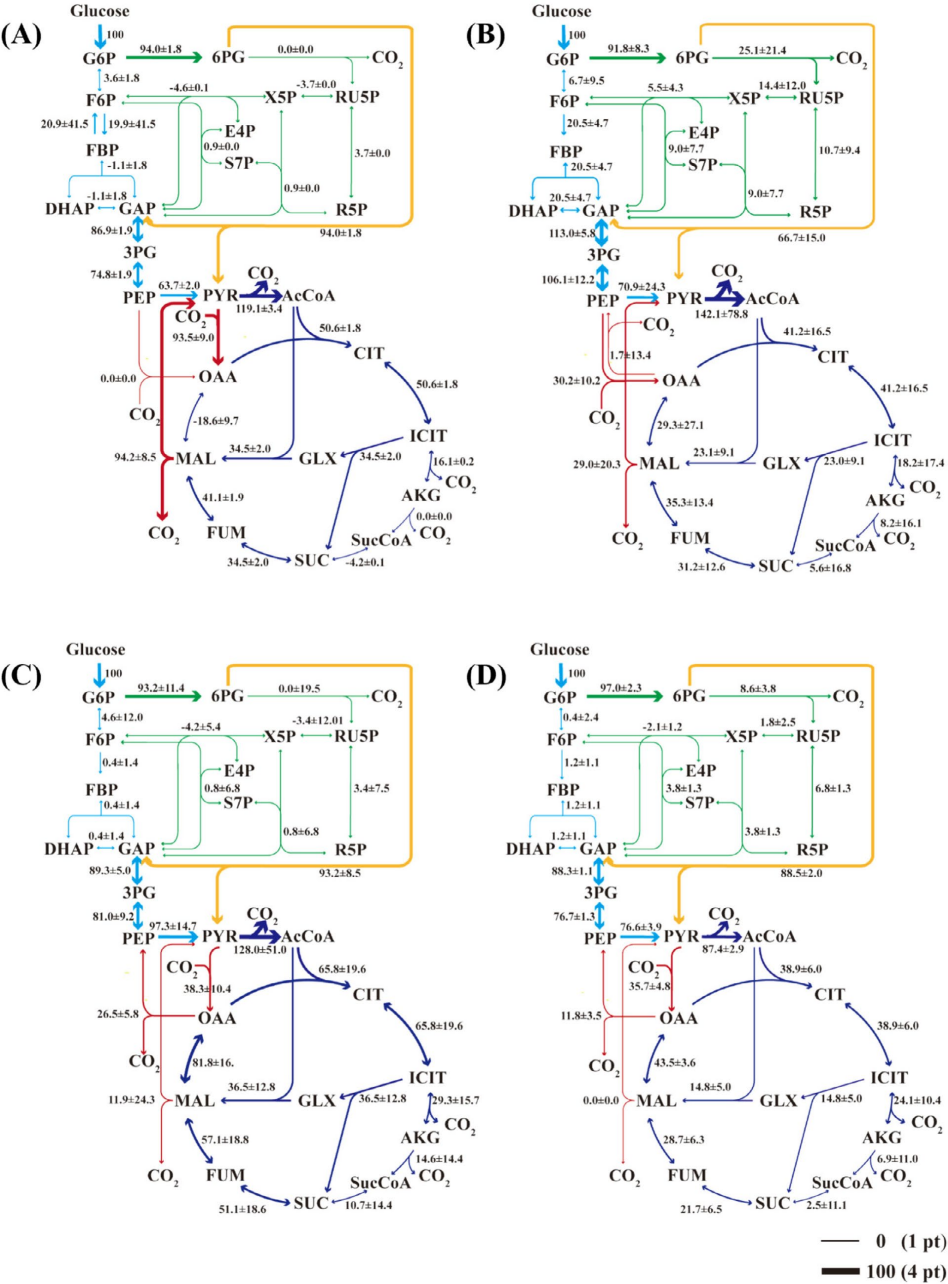
Based on ^13^C‐metabolic flux analysis with parallel ^13^C‐glucose isotopologues, the relative central carbon metabolic flux distribution in *S. marianensis* PS1 (A), *S. piezotolerans* WP3 (B), *G. profundi* WS11 (C) and *P. marina* W43 (D). It is estimated from the parallel ^13^C‐labelled treatments with a mixture of 1‐^13^C‐glucose (100%), U‐^13^C_6_‐glucose/natural glucose (20:80) and 1‐^13^C glucose/U‐^13^C_6_‐glucose (80:20). The full names of abbreviated metabolites refer to the Supporting Information.

The controlling factors of growth rates can be further elucidated by investigating intracellular metabolic flux patterns (Dominguez et al. 1998). Quantitative estimates of anabolic and catabolic activities using the ^13^C‐MFA approach revealed substantial differences in glucose utilisation fluxes among the four strains. Regarding the glycolytic pathways, both methods, i.e., alanine‐based and INCA flux modelling (see Section 2 for details), found that four strains primarily relied on the EDP for glucose utilisation (Figures 2 and 3).

However, the estimated flux values varied depending on the algorithms and the isotope labelling strategies applied (Crown and Antoniewicz 2013; Crown et al. 2015; Long and Antoniewicz 2019). The methodological framework applied in this study was motivated by Klingner et al. (2015), who first reported the fundamental metabolic flux patterns of marine bacteria. Using this approach, we observed that the EDP accounted for more than 80% of glucose catabolism in all four strains (Figure 2A). These values are comparable to those reported by Klingner et al. (2015), in which 22 out of 25 coastal bacterial strains predominantly utilised the EDP (81%–95%). The slightly lower EDP contribution observed in our strains may indicate metabolic differences between deep‐ocean and coastal marine bacteria. Moreover, the combination patterns of three glycolytic pathways varied among the four strains, with the EDP operating in parallel with either the EMPP or PPP. This pattern is consistent with the previous investigation of marine microbes isolated from the water column (Klingner et al. 2015), supporting that deep‐sea bacteria employ flexible glycolytic pathways to meet energy requirement. In addition, we observed a discrepancy between the two approaches particularly for PS1 and W43: the alanine‐based method suggested the coexistence of EMPP and EDP, whereas the INCA‐based analysis indicated PPP and EDP. This difference is likely due to the limitation of using a single tracer (1‐^13^C‐glucose) in the alanine‐based approach, which has reduced resolving power for distinguishing flux partitioning between EMPP and PPP (Leighty and Antoniewicz 2013). Moreover, this discrepancy highlights the potential for more diverse glycolytic strategies in marine bacterial strains. Importantly, this limitation does not affect the overall conclusion that EDP is the dominant glycolytic pathway in these strains.

The EDP dominance in marine bacteria is evidenced by the incubation of pure strains in this study, whereas the EDP does not appear to predominantly control glucose metabolism within sedimentary microbial communities, even though our isolates were obtained from a similar environment (e.g., oxygen depletion in sediment/surface interface). Recently it was hypothesised that the classic EMPP widely used in the terrestrial models (e.g., *E. coli* and *B. subtilis* ) (Fuhrer et al. 2005; Haverkorn van Rijsewijk et al. 2011) is preferred by sedimentary microbial communities (Hutchinson et al. 2024). The dominance of EMPP within sediment microbes contradicts the observation in our study and the previous investigation of microorganisms in the coastal seawater (Klingner et al. 2015). It could be attributed to the microbial interplay or metabolite exchange within community or the metabolic flux regulation in response to environmental alteration. Microorganisms preferring EDP, such as *S. marianensis* PS1 in this study, lack phosphofructokinase on the genome. As a result, strain PS1 cannot convert fructose‐6‐phosphate into fructose‐1,6‐bisphosphate for the complete EMPP (Nikel et al. 2015). In sediment microbial community, some microbial members have the complete EMPP and the generated fructose‐6‐phosphate would be further used by other groups that are depleted in phosphofructokinase. Remarkably, none of the four deep‐sea bacterial strains were annotated with phosphofructokinase in the KEGG database (Cao et al. 2020; Ding et al. 2019; Xiao et al. 2007; Yang et al. 2022; Zhang et al. 2022). Consistent with this, flux analysis indicates minimal carbon flow through the canonical EMPP pathway, suggesting that glycolysis via the classical EMPP route is not a major mode of carbohydrate catabolism in these organisms. The small inferred EMPP‐associated flux likely reflects reversible carbon exchange around the G6P–F6P node rather than a complete EMPP glycolytic pathway. Further studies are required to resolve the functional organisation of CCM in deep‐sea microbial communities.

### 3.3 Non‐Oxidative Pentose Phosphate Pathway in Genus *Shewanella*

*S. piezotolerans* WP3 exhibited distinct metabolic patterns compared with the other three strains. Specifically, *S. piezotolerans* WP3 decomposed glucose through the parallel operation of the EDP, PPP and EMPP. The PPP flux in this strain reached up to 25%, with a comparable contribution from both the oxidative phase (6PG → RU5P + CO_2_) and the non‐oxidative phase (RU5P → X5P, Figure 3B). Notably, the non‐oxidative PPP flux accounted for more than 50% of the total PPP input flux (14.4/25.1 = 57.4%; Figure 3B and Table 3), whereas the other strains showed almost no flux through the non‐oxidative phase of the PPP (Figure 3). The non‐oxidative phase of the PPP is an essential route for ribose‐5‐phosphate production, which supports key anabolic processes, such as riboflavin and DNA/RNA biosynthesis (Dauner et al. 2001). Riboflavin has been reported as a critical cofactor in anabolic Fe(III) reduction (Marsili et al. 2008; Zhao et al. 2023). The pronounced flux through the non‐oxidative PPP in *S. piezotolerans* WP3 therefore suggests a potential requirement for riboflavin to support alternative reductive pathways. In contrast, the negligible non‐oxidative PPP fluxes in the other three strains imply that riboflavin‐dependent reductive pathways may not be equally central to their physiology. However, future studies should clarify whether this trait is specific to *S. piezotolerans* WP3, e.g., by the chemical analysis of riboflavin.

**TABLE 3:** Metabolic flux ratios obtained by INCA analysis of experiments with parallel ^13^C‐labelled glucose.

Organisms	Mean metabolic flux (%) ± SD								
	6PG from G6P	PYR from 6PG	RU5P from 6PG	OAA from PEP	OAA from PYR	PYR from MAL	PEP from OAA	GLX from ICIT	Flux into TCA
*S. marianensis* PS1	94.0 ± 1.8	94.0 ± 1.8	0.0 ± 0.0	0.0 ± 0.0	93.5 ± 9.0	94.2 ± 8.5	n.a.	34.5 ± 2.0	50.6 ± 1.8
*S. piezotolerans* WP3	91.8 ± 8.3	66.7 ± 15.0	25.1 ± 21.4	30.2 ± 10.2	n.a.	29.0 ± 20.3	1.7 ± 13.4	23.0 ± 9.1	41.2 ± 16.5
*G. profundi* WS11	93.2 ± 11.4	93.2 ± 8.5	0.0 ± 19.5	n.a.	38.3 ± 10.4	11.9 ± 24.3	26.5 ± 5.8	36.5 ± 12.8	65.8 ± 19.6
*P. marina* W43	97.0 ± 2.3	88.5 ± 2.0	8.6 ± 3.8	n.a.	35.7 ± 4.8	0.0 ± 0.0	11.8 ± 3.5	14.8 ± 5.0	38.9 ± 6.0

Abbreviations: G6P, glucose‐6‐phosphate; GLX, glyoxylate; ICIT, isocitrate; MAL, malate; n.a., not applicable; OAA, oxaloacetate; PEP, phosphoenolpyruvate; PG, 6‐phosphoglucanate; PYR, pyruvate; RU5P, ribulose‐5 phosphate.

### 3.4 Equivalent Importance of Phosphoenolpyruvate vs. Pyruvate Carboxylation in Anaplerotic Reactions

Anaplerotic reactions serve as critical metabolic links between catabolism, anabolism and cellular energy metabolism by replenishing intermediates of the TCA cycle (Sauer and Eikmanns 2005). Two distinct routes of anaplerotic reactions were identified among the four deep‐sea bacterial strains studied here: pyruvate carboxylation and phosphoenolpyruvate (PEP) carboxylation. *S. marianensis* PS1, *G. profundi* WS11 and *P. marina* W43 primarily utilised pyruvate carboxylation, whereas *S. piezotolerans* WP3 favoured the PEP carboxylation (Figure 3). The choice of anaplerotic pathway appears to reflect the specific precursor demands of the TCA cycle, such as C4 metabolites oxaloacetate or malate, which are required for biosynthesis and energy balance (Figure 3). This flexible routing of carbon through anaplerotic reactions is expected to meet distinct physiological needs (Klingner et al. 2015).

Anaplerotic replenishment of the TCA cycle can be achieved via multiple routes, including PEP/pyruvate carboxylation and the glyoxylate shunt. The glyoxylate shunt bypasses CO_2_‐releasing steps of the TCA cycle and enables carbon conservation through the conversion of acetyl‐CoA into four‐carbon intermediates. In contrast, PEP and pyruvate carboxylation reactions directly incorporate inorganic carbon into central metabolism. Flux analysis indicates that both pathways are active in the studied strains, suggesting flexible strategies for TCA cycle maintenance under energy‐ and carbon‐limited deep‐sea conditions (Liu et al. 2022; Molari et al. 2013). Thus, the presence of alternative anaplerotic strategies likely enhances the metabolic resilience of deep‐sea bacteria by optimising carbon utilisation efficiency and maintaining TCA cycle homeostasis under nutrient‐limited conditions.

### 3.5 Metabolic Flux Regulation Against Oxidative Pressure

**FIGURE 4:**
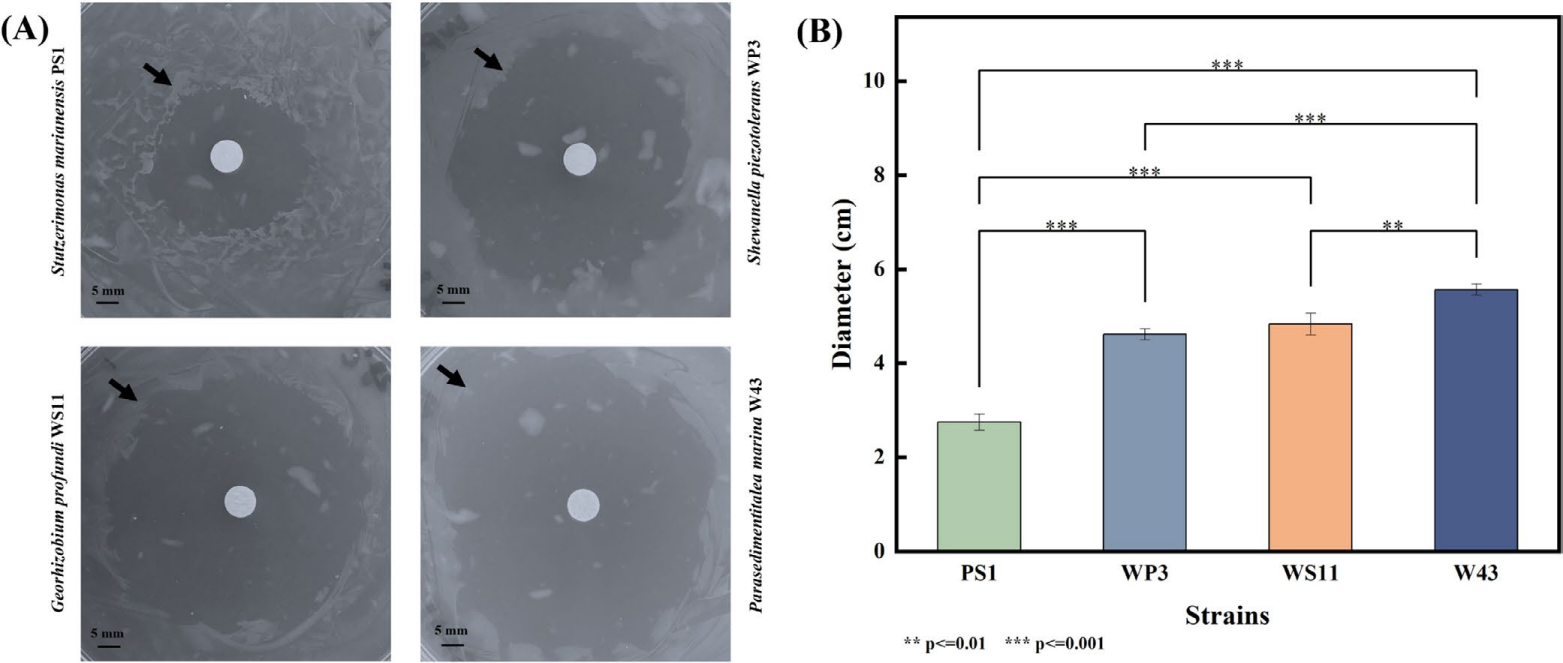
Evaluation of oxidative stress tolerance and its relationship with metabolic fluxes of cofactors. (A) Disk diffusion assay of four bacterial strains and (B) quantification of inhibition zone diameters.

The importance of the EDP in marine bacteria has been proposed to be associated with enhanced resistance to oxidative stress (Klingner et al. 2015; Xie et al. 2018). Previous studies suggested that the antioxidative ability is an important adaptive strategy in deep‐sea conditions, e.g., high hydrostatic pressure and low temperature (Xie et al. 2018). This raises the possibility that metabolic pathway selection may influence oxidative stress tolerance. To evaluate this relationship, we conducted an H_2_O_2_ antioxidative assay and compared these results with metabolic flux patterns (Figure 4A,B).

Compared to the EMPP, the EDP has lower ATP yield but reduced enzymatic burden, and it operates in conjunction with NADPH‐generating reactions in upper glycolysis and the pentose phosphate pathway (Flamholz et al. 2013). While the EMPP does not contribute directly to NADPH production, and the PPP can incur carbon loss, the EDP may enable a balanced metabolic state that supports both carbon efficiency and reducing power generation (Klingner et al. 2015; Rui et al. 2015; Singh et al. 2008).

Our analysis shows that antioxidative performance correlates strongly with NADPH supply flux, but not with intracellular NADPH concentration (Figure 1C), indicating that the rate of NADPH regeneration rather than pool size is critical for oxidative stress defence. Notably, intracellular ATP levels exhibit a significant negative correlation with antioxidative performance, suggesting that metabolic states characterised by lower ATP accumulation or higher ATP turnover are associated with improved oxidative stress tolerance. This pattern is consistent with a shift in cellular resource allocation away from ATP accumulation and towards redox homeostasis under oxidative stress (Rui et al. 2010).

In addition to metabolic factors, genomic analysis revealed substantial variation in the genetic potential for oxidative stress defence among the strains examined. The global oxidative stress regulator *OxyR* (Chiang and Schellhorn 2012) was conserved across all four strains, whereas genes encoding hydrogen peroxide‐detoxifying enzymes differed markedly. Strain PS1 possessed genes encoding *katE*, *katG*, *gpx* and *aphC*; strain WP3 harboured *katB*, *gpx* and *aphC*; whereas strains WS11 and W43 contained only *katG*. These differences in antioxidant gene repertoires likely play a major role in shaping strain‐specific antioxidative capacities and may outweigh the contribution of central carbon metabolic pathway preference to oxidative stress tolerance (Aertsen et al. 2005; Xie et al. 2018).

Together, these results suggest that oxidative stress tolerance in these deep‐sea bacteria is governed by an interplay between metabolic flux organisation and genetic capacity for detoxification. The predominance of the EDP may reflect a metabolic strategy that supports NADPH regeneration and flexible energy utilisation, while strain‐specific antioxidant systems further modulate the overall oxidative stress response.

## 4 Conclusions

In conclusion, heterotrophic microbes in the deepsea are expected to harbour diverse metabolisms to adapt to low organic matter, low temperature, and high hydrostatic pressure. In this study, we employed ^13^C‐MFA to investigate intracellular flux patterns through the CCM network in four bacterial strains isolated from surface sediment at different water depths. The dominance of the EDP as a primary glycolytic pathway for glucose degradation may represent a key strategy for microbial survival under oxidising conditions in benthic deep‐sea sediments, including those from the deepest regions of the Mariana Trench on Earth. The anaplerotic routes, involving pyruvate and phosphoenolpyruvate carboxylation, showed substantial diversity among the four strains, further providing additional carbon and energy inputs to the TCA cycle and gluconeogenesis. These metabolic traits likely help microbes compensate for carbon‐limited environments and enhance carbon utilisation efficiency. To our knowledge, this is the first time reporting the metabolic flux pattern for deep‐sea microbes, providing new insights into their dynamic metabolic processes and adaptive strategies.

## Author Contributions

**Yue Wu:** methodology, validation, investigation, funding acquisition, writing – review and editing, project administration. **Yue Li:** investigation. **Yuxue Yang:** writing – original draft, writing – review and editing, methodology, validation, investigation, software. **Keni Ma:** investigation. **Junwei Cao:** funding acquisition, validation, writing – review and editing. **Yuli Wei:** resources, writing – review and editing. **Ruilian Yao:** writing – review and editing, software. **Weichao Wu:** methodology, validation, funding acquisition, writing – original draft, writing – review and editing, project administration, supervision, resources, data curation, formal analysis, software.

## Funding

This work was funded by the National Key R&D Program of China (2024YFC2816200), the National Natural Science Foundation of China including Young Scientist Fund and General Program for W. Wu (42106046, 42376047) and J. Cao (41706146), the Natural Science Foundation of Shanghai (25ZR1402191) and the China Postdoctoral Science Foundation (2025M771291) for Y. Wu.

## Conflicts of Interest

The authors declare no conflicts of interest.

## Supporting information


**Figure S1:** Calibration curve for glucose concentrations (A), NADH (B) and NADPH (C). The analyses of these substrates were performed with commercial enzyme assay kits.
**Figure S2:** Statistical goodness‐of‐fit values of the selected amino acid fragments for metabolic flux modelling of four bacterial strains in this study. (A) PS1; (B) WP3; (C) WS11 and (D) W43. Acceptability of the fit was evaluated using a chi‐square test on the sum of squared residuals.
**Table S1:** Reactions involved in the metabolic network.

**Data S1:** emi470379‐sup‐0002‐DataS1.zip.

## Data Availability

All raw data, modelled data and summarised table are attached in the Supporting Information.
