# Supplementary material for: Metabolic Flux Analysis Reveals Entner–Doudoroff Pathway Dominance in Heterotrophic Deep‐Sea Bacterial Isolates
Source: Environ Microbiol Rep. 2026 Jun 12;18(3):e70379. doi: 10.1111/1758-2229.70379 (PMC13261371; doi:10.1111/1758-2229.70379)
Supplement: Supplementary file 1 — Figure S1: Calibration curve for glucose concentrations (A), NADH (B) and NADPH (C). The analyses of these substrates were performed with commercial enzyme assay kits. Figure S2: Statistical goodness‐of‐fit values of the selected amino acid fragments for metabolic flux modelling of four bacterial strains in this study. (A) PS1; (B) WP3; (C) WS11 and (D) W43. Acceptability of the fit was evaluated using a chi‐square test on the sum of squared residuals. Table S1: Reactions involved in the metabolic network. [file EMI4-18-e70379-s002.docx]

**Supporting information**

**Metabolic Flux Analysis Reveals Entner-Doudoroff Pathway Dominance in Heterotrophic Deep-sea Bacterial Isolates**

Yuxue Yang^1,2#^, Yue Wu^1,2#^, Keni Ma^1^, Yue Li^1^, Junwei Cao^1^, Yuli Wei^1^, Ruilian Yao^3^, Weichao Wu^1,2*^

1. College of Oceanography and Ecological Science, Shanghai Ocean University, Shanghai, China
2. International Research Center for Marine Biosciences at Shanghai Ocean University, Ministry of Science and Technology, China
3. State Key Laboratory of Microbial Metabolism, and School of Life Sciences and Biotechnology, Shanghai Jiao Tong University, Shanghai, China

^#^These authors contributed equally: Y. Yang, Y. Wu

*Corresponding author: wcwu@shou.edu.cn

***Short title***: Metabolic fluxes of deep-sea bacteria

**Measurement of glucose consumption rate, ATP, NAD^+^/NADH, and NADP^+^/NADPH:**

Cells of strains PS1, WP3, WS11, W43, and 168 were harvested at isotopic steady state with three biological replicates. Glucose consumption rates were determined from culture supernatants collected during the late exponential phase. Briefly, 1 mL of supernatant was filtered through a 0.22 µm PES membrane (25 mm), and glucose concentrations were quantified using a commercial assay kit (AKSU001, Boxbio, China) according to the manufacturer’s instructions, with absorbance measured at 505 nm. Glucose concentrations were calculated using a standard curve (Fig. S1A).

The glucose consumption rate was calculated as the difference between the initial glucose concentration and the concentration at the sampling time, divided by the incubation time.

Glucose consumption rate = (C_initial − C_t) / Δt

where C_initial and C_t represent glucose concentrations at initial and sampling time points, respectively. Δt is the incubation time between the initial and sampling time points.

Intracellular ATP, NAD⁺/NADH, and NADP⁺/NADPH levels were measured during the late exponential phase. Cells were harvested by centrifugation at 12,000 × g for 10 min at 4 °C. For ATP analysis, approximately 5 × 10⁶ cells were collected, whereas for NAD⁺/NADH and NADP⁺/NADPH measurements, culture volumes corresponding to 1.25 OD₆₀₀ units were used. In addition, these metabolites were measured in *B. subtilis* as a reference organism for comparative analysis, following a previously reported protocol (Klingner et al. 2015).

For ATP measurement, cell pellets were resuspended in 1 mL of extraction buffer and lysed by ultrasonic disruption in an ice bath (200 W; 3 s pulses with 10 s intervals, repeated 30 times). The lysate was centrifuged at 12,000 × g for 10 min at 4 °C, and the supernatant was collected for analysis. ATP was quantified with an enzymatic reaction coupled to hexokinase and glucose-6-phosphate dehydrogenase, in which ATP-dependent phosphorylation leads to the production of NADPH. The generated NADPH was measured spectrophotometrically at 340 nm. This assay was performed using a commercial kit (BL852A, Biosharp, China) according to the manufacturer’s instructions, and ATP concentrations were calculated based on the provided conversion equation.

ATP content (μmol/10⁴ cells) = [ΔA ÷ (ε×L) × V2 × 10⁶] ÷ (500 × V1 ÷ V)

where ΔA is the absorbance after subtraction of the blank; ε is the molar extinction coefficient of NADPH (6.3 × 10³ L·mol⁻¹·cm⁻¹); *L* is the optical path length (cm, 1 cm); V is the total extraction volume (1 mL); V₁ is the volume of sample used in the assay; and V₂ is the total reaction volume.

For NAD⁺/NADH and NADP⁺/NADPH measurements, cell pellets were resuspended in 200 µL of the corresponding pre-chilled extraction buffer and gently mixed by pipetting, followed by centrifugation at 12,000 × g for 10 min at 4 °C. The resulting supernatants were collected for analysis. NAD⁺/NADH and NADP⁺/NADPH were quantified using WST-8–based enzymatic assays, in which NADH and NADPH reduce WST-8 via electron coupling reactions catalyzed by alcohol dehydrogenase and glucose-6-phosphate dehydrogenase, respectively. Absorbance was measured at 450 nm using a UV–Vis spectrophotometer. Assays were performed using commercial kits (NAD⁺/NADH: S0175; NADP⁺/NADPH: S0179; Beyotime, China) according to the manufacturer’s instructions, and concentrations were determined based on standard curves (Fig. S1B and S1C)

**Metabolic flux analysis**

The experimental and simulated mass distribution vectors (MDVs) for the four strains are shown in Fig. S2. The acceptable range of SSR values is defined between χ^2^_α/2_ (*n*–*p*) and χ^2^_1-α/2_ (*n*–*p*), where α is the chosen significance level (0.05 in this study), n is the number of fitted measurements (i.e., the total numbers of isotopomers), and *p* is the number of estimated independent parameters (i.e., fluxes).

A good quality of fit was obtained for all strains, with sum of squared residuals (SSR) values of 299.7, 232.0, 283.3, and 244.7 for strains PS1, WP3, WS11, and W43, respectively (Fig. S2). These values fall within the expected χ² ranges at the 95% confidence level (219.1–308.7 for PS1, 160.9–238.9 for WP3, 224.5–315.2 for WS11, and 243.8–338.0 for W43), indicating that all models provide statistically acceptable fits to the experimental data.

**List of Metabolite Abbreviations**

3PG/PG3, 3-phosphoglycerate; 6PG/PG6, 6- phosphogluconate; Ac, acetate; AcCoA, acetyl-CoA; AKG, α-ketoglutarate; Ala, Alanine; Arg, Arginine; Asn, Asparagine; Asp, Aspartate; ATP, adenosine triphosphate; CIT, citrate; CO2, carbon dioxide; Cys, Cysteine; DHAP, dihydroxyacetone phosphate; E4P, erythrose-4-phosphate; F6P, fructose-6 phosphate; FADH2, flavin adenine dinucleotide; FBP, fructose-1,6-bisphosphate; FTHF, formyltetrahydrofolate; FUM, fumarate; G6P, glucose-6-phosphate; GAP, glyceraldehyde-3 phosphate; Gluc.ext, glucose; Gly, glycine; Gln, glutamine; Glu, glutamate; Glyox/GLX, glyoxylate; His, Histidine; ICIT, isocitrate; Ile, isoleucine; KDPG, 2-dehydro-3-deoxy-phosphogluconate; Leu, Leucine; LL_DAP, l,l-diaminopimelate; Lys, Lysine; MAL, malate; Met, methionine; MEETHF, methylene-tetrahydrofolate; METHF, methyl-tetrahydrofolate; NADPH, nicotinamide adenine dinucleotide phosphate; NADH, nicotinamide adenine dinucleotide; OAA, oxaloacetate; PEP, phosphoenolpyruvate; Phe, phenylalanine; Pro, proline; R5P, ribose-5-phosphate; Ru5P, ribulose-5 phosphate; PYR, pyruvate; S7P, sedoheptulose-7-phosphate; Ser, Serine; SUC, succinate; SucCoA, succinyl-CoA; Thr, Threonine; Tyr, Tyrosine; Trp, tryptophan; Val, Valine; X5P, xylulose-5-phosphate.

**
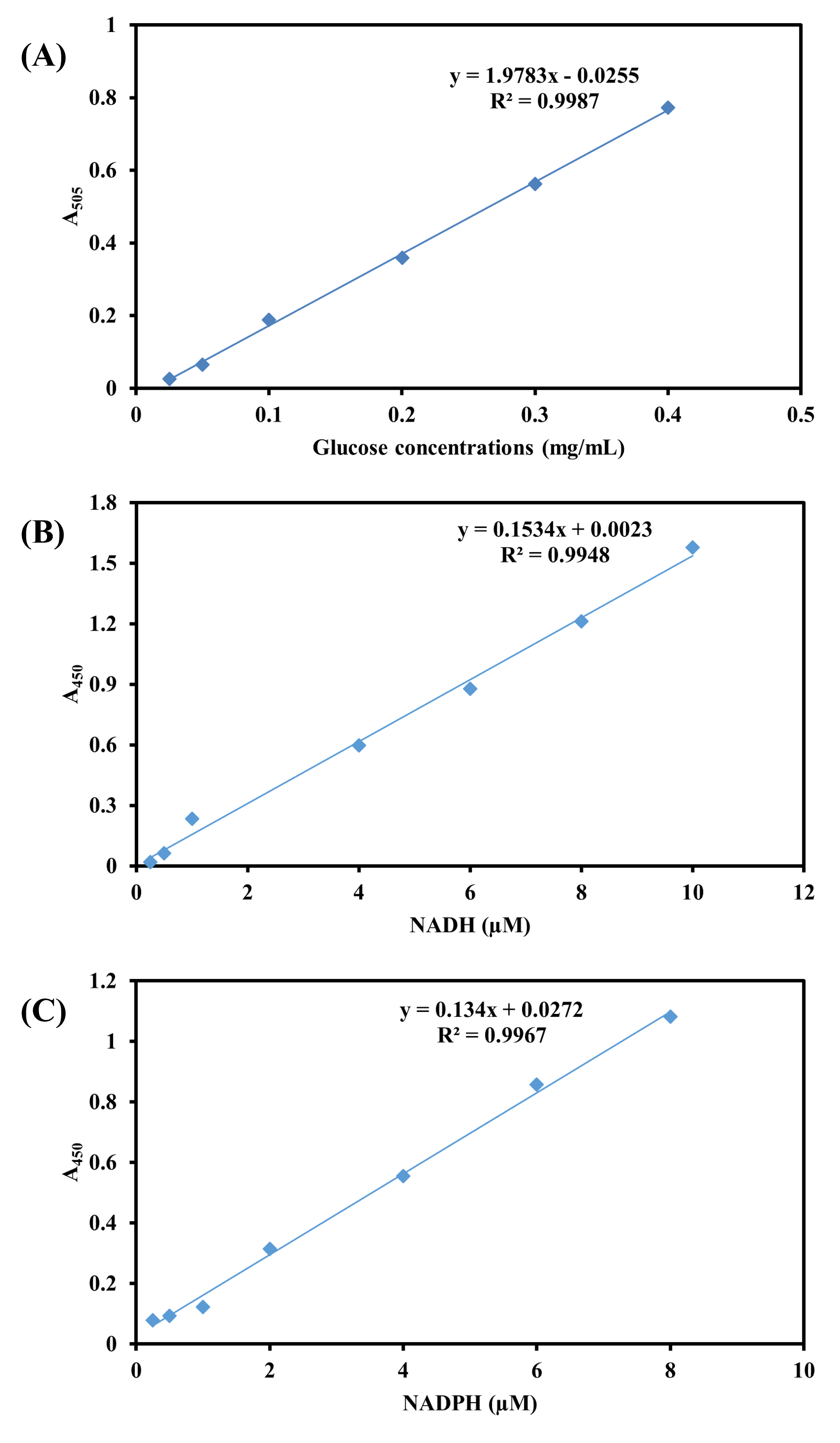
**

**Fig. S1** Calibration curve for glucose concentrations (A), NADH (B) and NADPH (C). The analyses of these substrates were performed with commercial enzyme assay kits.


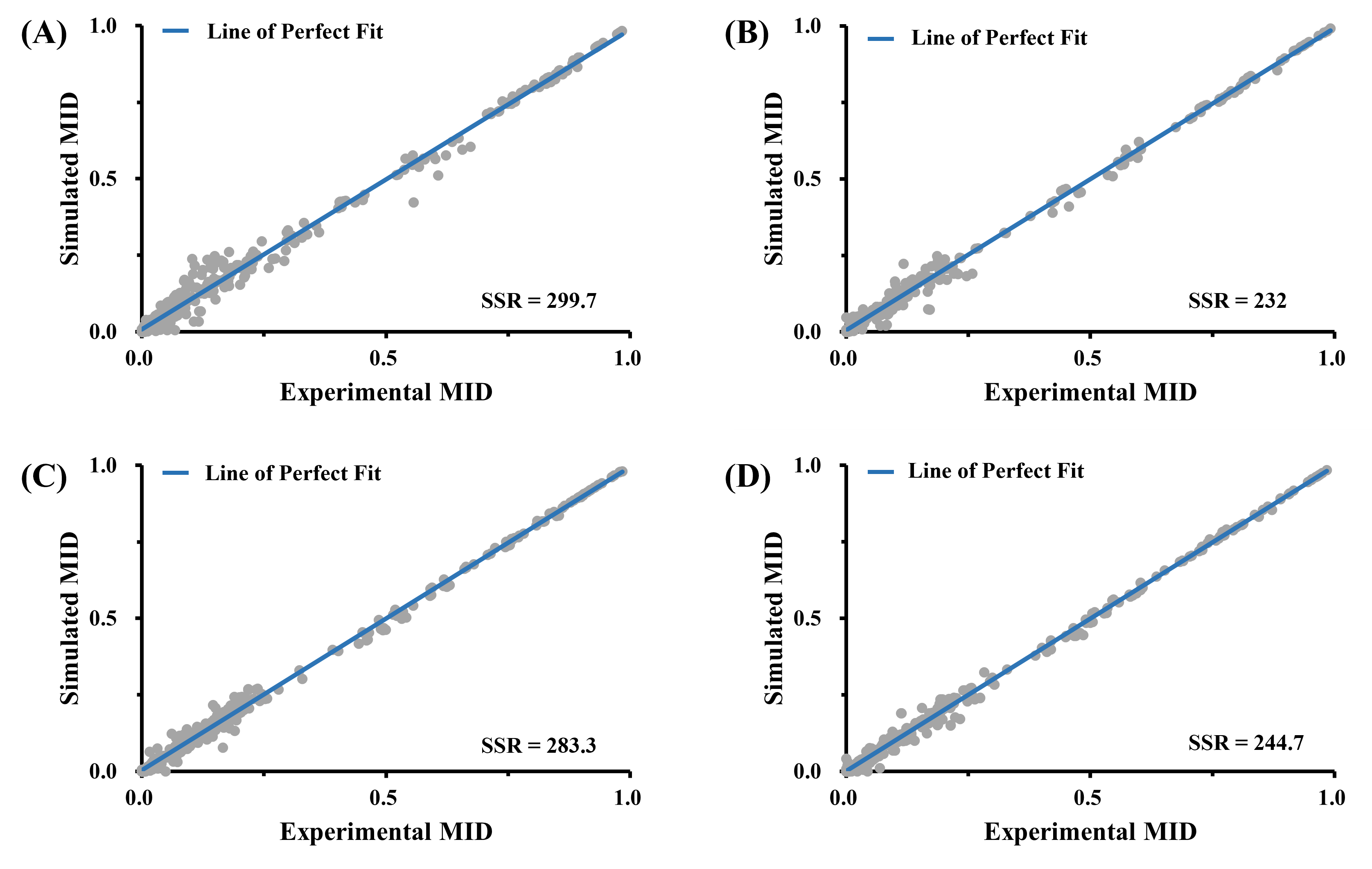


**Fig. S2** Statistical goodness-of-fit values of the selected amino acid fragments for metabolic flux modelling of four bacterial strains in this study. A, PS1; B, WP3; C, WS11; D, W43. Acceptability of the fit was evaluated using a chi-square test on the sum of squared residuals.

**Table S1. Reactions involved in the metabolic network**

| Glycolysis | Reactions |
| --- | --- |
| v_1_  v_2_  v_3_  v_4_  v_5_  v_6_  v_7_  v_8_  v_9_ | Gluc.ext (abcdef) + ATP -> G6P (abcdef)  G6P (abcdef) <-> F6P (abcdef)  F6P (abcdef) + ATP -> FBP (abcdef)  FBP (abcdef) -> F6P (abcdef) + ATP  FBP (abcdef) <-> DHAP (cba) + GAP (def)  DHAP (abc) <-> GAP (abc)  GAP (abc) <-> PG3 (abc) + ATP + NADH  PG3 (abc) <-> PEP (abc)  PEP (abc) -> Pyr (abc) + ATP |
| Pentose Phosphate Pathway | |
| v_10_  v_11_  v_12_  v_13_  v_14_  v_15_  v_16_ | G6P (abcdef) -> PG6 (abcdef) + NADPH  PG6 (abcdef) -> Ru5P (bcdef) + CO2 (a) + NADPH  Ru5P (abcde) <-> X5P (abcde)  Ru5P (abcde) <-> R5P (abcde)  R5P (abcde) + X5P (fghij) <-> S7P (fgabcde) + GAP (hij)  E4P (abcd) + X5P (efghi) <-> F6P (efabcd) + GAP (ghi)  S7P (abcdefg) + GAP (hij) <-> E4P (defg) + F6P (abchij) |
| Entner-Doudoroff Pathway | |
| v_17_  v_18_ | PG6 (abcdef) -> KDPG (abcdef)  KDPG (abcdef) -> Pyr (abc) + GAP (def) |
| TCA Cycle | |
| v_19_  v_20_  v_21_  v_22_  v_23_  v_24_ | Pyr (abc) -> AcCoA (bc) + CO2 (a) + NADH  OAA (abcd) + AcCoA (ef) -> Cit (dcbfea)  Cit (abcdef) <-> ICit (abcdef)  ICit (abcdef) <-> AKG (abcde) + CO2 (f) + NADPH  AKG (abcde) -> SucCoA (bcde) + CO2 (a) + NADH  SucCoA (abcd) <-> Suc (abcd) + ATP |
| v_25_  v_26_  v_27_ | Suc (abcd) <-> Fum (abcd) + FADH2  Fum (abcd) <-> Mal (abcd)  Mal (abcd) <-> OAA (abcd) + NADH |
| Glyoxylate Shunt | |
| v_28_  v_29_ | ICit (abcdef) -> Glyox (ab) + Suc (edcf)  Glyox (ab) + AcCoA (cd) -> Mal (abdc) |
| Anaplerotic Reactions | |
| v_30_  v_31_  v_32_  v_33_ | Mal (abcd) -> Pyr (abc) + CO2 (d) + NADPH  PEP (abc) + CO2 (d) -> OAA (abcd)  Pyr (abc) + CO2 (d) + ATP -> OAA (abcd)  OAA (abcd) + ATP -> PEP (abc) + CO2 (d) |
| Acetate formation | |
| v_34_ | AcCoA (ab) <-> Ac (ab) + ATP |
| Amino Acid Biosynthesis | |
| v_35_  v_36_  v_37_  v_38_  v_39_  v_40_  v_41_  v_42_  v_43_  v_44_  v_45_  v_46_  v_47_  v_48_ | AKG (abcde) + NADPH -> Glu (abcde)  Glu (abcde) + ATP -> Gln (abcde)  Gln (abcde) + AKG (fghij) + NADPH -> Glu (abcde) + Glu (fghij)  Glu (abcde) + ATP + 2*NADPH -> Pro (abcde)  Glu (abcde) + CO2 (f) + Gln (ghijk) + Asp (lmno) + AcCoA (pq) + 5*ATP + NADPH -> Arg (abcdef) + AKG (ghijk) + Fum (lmno) + Ac (pq)  OAA (abcd) + Glu (efghi) -> Asp (abcd) + AKG (efghi)  Asp (abcd) + 2*ATP -> Asn (abcd)  Pyr (abc) + Glu (defgh) -> Ala (abc) + AKG (defgh)  PG3 (abc) + Glu (defgh) -> Ser (abc) + AKG (defgh) + NADH  Ser (abc) <-> Gly (ab) + MEETHF (c)  Gly (ab) <-> CO2 (a) + MEETHF (b) + NADH  Thr (abcd) -> Gly (ab) + AcCoA (cd) + NADH  Ser (abc) + AcCoA (de) + 3*ATP + 4*NADPH -> Cys (abc) + Ac (de)  Asp (abcd) + Pyr (efg) + Glu (hijkl) + SucCoA (mnop) + 2ATP + |
| v_49_  v_50_  v_51_  v_52_  v_53_  v_54_  v_55_  v_56_  v_57_  v_58_ | 2*NADPH -> LL_DAP (abcdgfe) + AKG (hijkl) + Suc (mnop)  LL_DAP (abcdefg) -> Lys (abcdef) + CO2 (g)  Asp (abcd) + 2*ATP + 2*NADPH -> Thr (abcd)  Asp (abcd) + METHF (e) + Cys (fgh) + SucCoA (ijkl) + ATP + 2*NADPH -> Met (abcde) + Pyr (fgh) + Suc (ijkl)  Pyr (abc) + Pyr (def) + Glu (ghijk) + NADPH -> Val (abcef) + CO2 (d) + AKG (ghijk)  AcCoA (ab) + Pyr (cde) + Pyr (fgh) + Glu (ijklm) + NADPH -> Leu (abdghe) + CO2 (c) + CO2 (f) + AKG (ijklm) + NADH  Thr (abcd) + Pyr (efg) + Glu (hijkl) + NADPH -> Ile (abfcdg) + CO2 (e) + AKG (hijkl)  PEP (abc) + PEP (def) + E4P (ghij) + Glu (klmno) + ATP + NADPH -> Phe (abcefghij) + CO2 (d) + AKG (klmno)  PEP (abc) + PEP (def) + E4P (ghij) + Glu (klmno) + ATP + NADPH -> Tyr (abcefghij) + CO2 (d) + AKG (klmno) + NADH  Ser (abc) + R5P (defgh) + PEP (ijk) + E4P (lmno) + PEP (pqr) + Gln (stuvw) + 3*ATP + NADPH -> Trp (abcedklmnoj) + CO2 (i) + GAP (fgh) + Pyr (pqr) + Glu (stuvw)  R5P (abcde) + FTHF (f) + Gln (ghijk) + Asp (lmno) + 5*ATP -> His (edcbaf) + AKG (ghijk) + Fum (lmno) + 2*NADH |
| One carbon metabolism | |
| v_59_  v_60_ | MEETHF (a) + NADH -> METHF (a)  MEETHF (a) -> FTHF (a) + NADPH |
| Transport |  |
| v_61_  v_62_ | Ac (ab) -> Ac.ext (ab)  CO2 (a) -> CO2.ext (a) |
| Transhydrogenation | |
| v_63_ | NADH <-> NADPH |
| Oxidative Phosphorylation | |
| v_64_  v_65_ | NADH -> 3*ATP  FADH2 -> 2*ATP |
| ATP Hydrolysis | |
| v_66_ | ATP -> ATP_maintenance |
| Biomass Formation | |
| v_67_ | 0.174*G6P + 0.068*F6P + 0.107*GAP + 1.882*AcCoA + 0.431*Gly + 0.263*Pro + 0.598*Ala + 0.389*Val + 0.628*Leu + 0.244*Ile + 0.122*Met + 0.055*Cys + 0.191*Phe + 0.135*Tyr + 0.077*Trp + 0.126*His + 0.18*Lys + 0.354*Arg + 0.251*Gln + 0.158*Asn + 0.301*Glu + 0.284*Asp + 0.301*Ser + 0.256*Thr + 46.75*ATP -> bio |
| Model constraints: v_31_ was inactive in strains WS11 and W43; v_32_ was inactive in strain WP3; v_33_ was inactive in strain PS1; v_37_ was active only in strain PS1. | |
